# Metallic Impurities in Canned Meats

**Published:** 1884-07

**Authors:** 


### Metallic Impurities in Canned Meats.

Dr. E. Davies (*Pharm. Jour. and Trans.*), says that his attention was recently called to a case of poisoning from eating tinned salmon, the doctor who had charge of the case having attributed the same to tin nitrate. Of course the chemistry of the medical gentleman was, to say the least, peculiar. Tin nitrate, either stannic or stannous, can only be formed by the action of nitric acid, either on tin or stannous hydrate for stannous nitrate, or on stannic hydrate for stannic nitrate. But there is no nitric acid in the flesh of salmon, and the formation of nitric acid from its nitrogen in the absence of oxygen is a chemical impossibility. Indeed, nitrates of tin are such unstable salts that they could not have endured the heat of preparing the tinned salmon, even if they had been purposely added. Whether tin can be taken up by the oil in which the salmon is cooked seemed, however, a possible thing, forming a fatty salt of tin. He, therefore, analyzed the oil from a tin of preserved salmon, using the cheapest kind. Three hundred grains gave .03 grain of residue on igniting the precipitated sulphides, of which a part was lead and a trace of tin. This amount is insufficient to cause any effect.

Whether tin in such a form is poisonous is not known. The only tin salts which have been known to possess poisonous properties are the stannous and stannic chloride. Stannic chloride, as usually prepared, contains free acid, and its strongly irritant effects may be thus partly accounted for, but these salts are not present in tinned meats. Tin in the metallic form is not at all poisonous, and tinned vessels have been in such constant use for cooking purposes that some cases must have been met with if they could impart deleterious properties to the food. The lead which is almost always present in the tin used for tinning may be supposed to be the active agent. He analyzed tinned beef for this metal and found on the whole of the outside of the meat in a 4-pound tin .07 of a grain. The interior of the meat was quite free from tin. Lead poisoning from small doses does not cause sudden symptoms. Paralysis is the usual result, and not symptoms of irritant poisoning.

The amount above stated is quite insufficient to produce any visible effect, and the removal of the outside would remove any that might be present. He also analyzed a tin of tomatoes which had apparently suffered partial decomposition. Two ounces gave .10 of residue when the sulphides were ignited. This was principally tin, with a minute amount of lead. The tomatoes were, of course, acid, and acid substances should not be preserved in tinned vessels, but even this quantity is so small that he should hesitate to assume that it could be injurious. The bad effects which have in a few cases resulted from the use of tinned meats are due to decomposition of the meat, owing to imperfect closing or faults in preparation. The results of his examination are that lead should be carefully excluded in the preparation of the tin plate, and that acid liquids should not be kept in tinned vessels, but that no cases are known in which any injurious effects have been proved to have resulted from metallic impurities in tinned meats.

